# Longitudinal Assessment of Large Optic Discs in Children and Adults Using Spectral Domain Optical Coherence Tomography

**DOI:** 10.3390/jcm15093348

**Published:** 2026-04-28

**Authors:** Julia V. Stingl, Sara Walter, Cordula Braun, Simon C. König, Dominik Wolters, Jasmin Rezapour, Felix M. Wagner, Achim Fieß, Alexander K. Schuster, Esther M. Hoffmann

**Affiliations:** Department of Ophthalmology, University Medical Center, Johannes Gutenberg-University Mainz, 55131 Mainz, Germany; stinglju@uni-mainz.de (J.V.S.);

**Keywords:** large optic discs, childhood glaucoma, optical coherence tomography, optic disc morphology, glaucoma

## Abstract

**Objectives**: Children with large optic discs are frequently referred to as glaucoma suspects. The aim of this study was to longitudinally analyze and compare the peripapillary retinal nerve fiber layer (pRNFL) thickness and minimum rim width (BMO-MRW) of large optic discs in children (LOD-C), normal sized optic discs in children (NOD-C) and large optic discs in adults (LOD-A) in order to evaluate longitudinal structural stability and intergroup differences in optic nerve head morphology. **Methods**: Briefly, 85 LOD-C, 72 NOD-C and 78 LOD-A were included. Large optic discs were identified based on a Bruch’s membrane opening (BMO) area threshold of ≥2.5 mm^2^. pRNFL thickness and minimal rim width (BMO-MRW) of optical coherence tomography (OCT) were compared between baseline and follow-up examination at a median of 1.4 [1.0; 2.5] years. **Results**: Mean global pRNFL thickness and BMO-MRW did not significantly change during the follow-up period. LOD-C showed a significant increase in pRNFL thickness in the superonasal segments, NOD-C in the nasal segments. pRNFL and BMO-MRW of LOD-A decreased during the follow-up period. However, there was a higher variance of pRNFL thickness change in LOD-C as compared with NOD-C in the superotemporal and inferonasal segments. Four optic discs in the LOD-A group showed a glaucomatous conversion, whereas none of the discs of LOD-C or NOD-C progressed to glaucoma. **Conclusions**: Children with large optic discs presented with stable pRNFL and BMO-MRW and without clinical progression to glaucoma at follow-up. A careful interpretation of the OCT measurement results is necessary when assessing pediatric large optic discs, as growth and remodelling may impair the accuracy of segmentation and automated classification algorithms.

## 1. Introduction

Large optic discs are frequently diagnosed as glaucoma suspects or even glaucomatous optic discs. Albeit histological studies found a higher neuroretinal nerve fiber count in large optic discs, the glaucomatous appearance is most likely explained by a similar amount of retinal nerve fibers distributing over a larger disc area, leading to a larger cup-to-disc ratio [1,2]. In children, visual field examination may be challenging and requires certain training to achieve reliable results [3]. Thus, optical coherence tomography (OCT) of the peripapillary retinal nerve fiber thickness (pRNFL) and Bruch’s membrane opening-minimum rim width (BMO-MRW) is commonly applied to rule out glaucoma; however, the specific anatomy of pediatric large optic discs deviates from the normative database’s optic discs, and automated algorithms frequently reach their limits [4].

We recently published a cross-sectional study comparing large optic discs in children (LOD-C) with normal-sized optic discs in children (NOD-C) and with large optic discs in adults (LOD-A) and found a positive correlation between BMO area and pRNFL thickness and a negative correlation between BMO area and BMO-MRW. pRNFL thickness over-proportionally increased in eyes with BMO area ≥ 2.8 mm^2^, while BMO-MRW in eyes with a BMO-Area ≤ 1.9 mm^2^. The analysis of the automated classification algorithm showed a higher frequency of borderline or outside normal limits classifications in the inferotemporal and superonasal pRNFL segments in the LOD-C group compared with the NOD-C group. This may reflect a heterogeneous arrangement of retinal nerve fibers and could potentially mislead the ophthalmologist toward diagnosing a glaucoma-suspect optic disc, despite the use of an objective and reliable measurement tool [4,5].

Besides determining whether glaucoma is present in eyes with large optic discs, an additional concern is whether these eyes exhibit an increased susceptibility to glaucomatous damage, a topic that remains controversial in adults [6,7,8,9,10]. While pediatric large optic discs have been shown to have a thicker neuroretinal rim, adult large optic discs demonstrate a thinner rim compared with normal controls, suggesting an accelerated loss of neuroretinal tissue over time [11]. To address this issue, we longitudinally followed participants from our previous cross-sectional study to evaluate the stability of large optic discs during childhood.

## 2. Materials and Methods

### 2.1. Participants

This was a retrospective longitudinal cohort study performed at the Department of Ophthalmology of the University Medical Center Mainz in Germany. Children with large optic discs (LOD-C), children with normal-sized optic discs (NOD-C) and adults with large optic discs (LOD-C) were identified by chart review. Patients referred as glaucoma suspects who turned out to be healthy or (in case of NOD-C) with other diagnoses that had not affected the optic nerve (such as strabism; a detailed list was previously published by Stingl JV et al. [4] with at least two OCT examinations were eligible for analysis. For baseline analysis, the cases were matched by age and sex (children groups), respectively, BMO area and sex (large optic discs groups) as described in our former publication [4]. Exclusion criteria comprised a history of glaucoma, defined by fundoscopic optic nerve head abnormalities (focal or diffuse rim thinning, defects in retinal nerve fiber layer, or asymmetric excavation) in combination with corresponding functional and OCT findings. Furthermore, patients under antiglaucomatous treatment or with ocular or cerebral disorders potentially affecting pRNFL thickness were not included. The control group consisted of individuals who had been referred for suspected ocular pathology (e.g., a glaucoma-suspect optic disc) that was subsequently excluded following comprehensive ophthalmologic evaluation. After a thorough assessment, these subjects were considered healthy, apart from mild refractive errors. The referral diagnoses, methods and results are described elsewhere [4]. In accordance with the traditional definition of megalopapilla, an optic disc area of or exceeding 2.5 mm^2^ was considered indicative of a large optic disc [2,11,12,13,14,15,16]. In the pre16sent study, this criterion was applied to OCT-derived BMO area measurements. Of these, all cases with a follow-up examination were included in this study.

### 2.2. Optical Coherence Tomography

The optic nerves were examined using Spectralis OCT (Heidelberg Engineering, Heidelberg, Germany) and the Glaucoma Module Premium Edition software (version 6.16.7.0). It provides 24 high-resolution radial optic nerve head (ONH) scans and 3 circle RNFL scans with a diameter of 3.5 mm, 4.1 mm and 4.7 mm, centered at the optic disc. Follow-up scans were acquired using the automatic follow-up mode of Spectralis OCT, which employs active eye tracking and exact scan positioning to ensure precise relocation of the baseline scan and identical B-scan placement across visits.

A systematic quality assessment of all OCT scans was carried out. Whenever the automated segmentation of the BMO, inner limiting membrane, or RNFL provided by the device software was inaccurate, the deviations were manually adjusted by JVS and SW. For scans showing more pronounced segmentation errors, a glaucoma specialist with certified OCT reading-center expertise (JR) reviewed and corrected the segmentation (number of eyes with corrected scans: LOD-C 27 (30.0%); NOD-C 6 (8.3%); LOD-A 7 (8.8%)). Scans were excluded from the analysis if the automated segmentation failed and a reliable manual correction could not be achieved in two or more images for BMO acquisition or in one circle of RNFL or BMO-MRW measurement.

### 2.3. Ophthalmological Assessment

Refractive error was measured using OCULUS/Nidek AR-1s (OCULUS, Wetzlar, Germany), and ocular biometry was obtained by IOL Master 700 (Zeiss Meditec, Jena, Germany). Visual fields were assessed using either Octopus 900 (Haag-Streit AG, Koenitz, Switzerland) or Humphrey Field Analyzer (Carl Zeiss Meditec, Jena, Germany). Intraocular pressure was obtained by Goldmann applanation tonometry (Haag-Streit AG, Koenitz, Switzerland) or iCare rebound tonometry (iCare IC200, Icare Finland Oy, Vanta, Finland). In case of low compliance, single instrumental examinations were skipped.

### 2.4. Glaucoma Assessment

Funduscopic optic nerve head evaluation was performed by glaucoma experts (JVS, JR). In case of significant thinning of pRNFL thickness in OCT with corresponding narrowing/notch of neuroretinal rim typical for glaucoma, and (in case of a reliable examination) corresponding visual field defects, a diagnosis of glaucoma was marked in the documents. Cases with diffuse, non-typical pRNFL thinning without glaucomatous optic nerve cupping or corresponding visual field defects were classified as glaucoma suspects.

Eyes with glaucoma or glaucoma suspect results were randomized with healthy eyes and underwent a re-evaluation of OCT and visual fields by two independent senior glaucoma experts (AKS, EMH), who were masked to the diagnosis.

### 2.5. Statistical Analysis

For statistical analysis was performed using R Studio (Version 2025.05.1+513). Means and standard deviations are provided for normally distributed variables, and medians and interquartile ranges (IQR) for nonnormally distributed variables. Paired *t*-tests were calculated to find differences in pRNFL thickness and BMO-MRW between baseline and follow-up examinations. Bonferroni’s correction was applied for multiple testing and an alpha < 0.007 was considered statistically significant. Furthermore, the differences between both measurements were illustrated using both boxplots and pRNFL plots (depicting each of the 768 single measurements). Because both eyes could be included in the analysis, the difference in pRNFL change between the groups was analyzed using mixed models with generalized estimating equations (GEE). Variance analysis of RNFL and MRW change was performed using the Brown-Forsythe test and Bonferroni correction (alpha < 0.007) to identify segments with a high variability between baseline and follow-up examinations.

## 3. Results

Of the initial 127 eyes per group [4], 85 (LOD-C), 72 (NOD-C) and 78 (LOD-A) eyes were available for inclusion for the follow-up analysis. The median follow-up time was 1.4 [1.0; 2.5] years over all three groups. Descriptive statistics at baseline are provided in Table 1.

To find changes in the pRNFL thickness and BMO-MRW segments, paired *t*-tests were performed. The mean differences and 95% confidence intervals are presented in Table 2. A significant increase in pRNFL thickness was present in the nasal superior segments and the nasal segments measured at 3.5 mm diameter in the pediatric groups; however, when Bonferroni’s correction was applied, only an increase in the nasal superior segments at 3.5 mm diameter and at 4.1 mm diameter in the LOD-C group, and an increase in the nasal segment at 3.5 mm in the NOD-C group remained statistically significant. Conversely, the adults’ pRNFL thickness slightly decreased during the follow-up period in the temporal superior segment at 3.5 and 4.1 mm diameter; this difference was not statistically significant after Bonferroni’s correction. BMO-MRW showed a statistically significant reduction with adult large optic discs in the temporal inferior, nasal inferior, nasal and nasal superior segments, remaining significant after Bonferroni’s correction.

**Figure 1:**
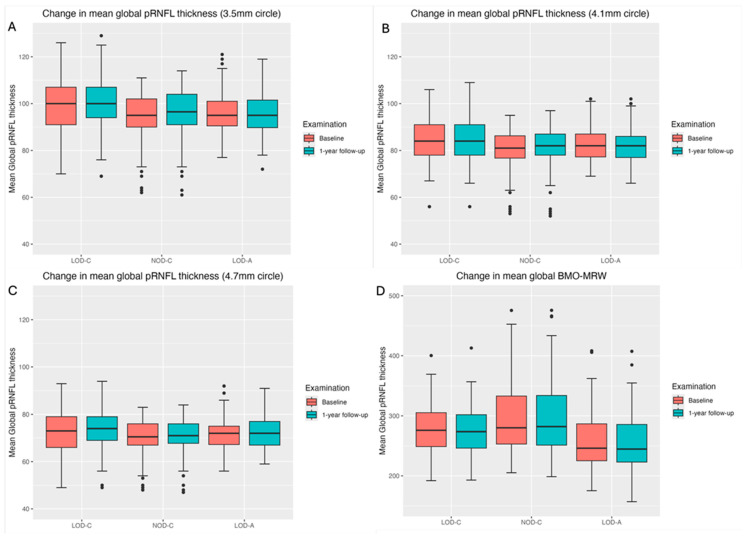
Boxplots of mean global pRNFL thickness (**A**–**C**) and mean global BMO-MRW (**D**) for LOD-C, NOD-C and LOD-A at baseline (orange) and follow-up (aqua).

Figure 1 shows a stable median thickness for global pRNFL in the circles with 3.5, 4.1 and 4.7 mm, global BMO-MRW at baseline and follow up. With respect to the segments, Appendix A.1 illustrates a higher variance of pRNFL thickness in children with large optic discs in the temporal superior, nasal superior, temporal inferior and nasal inferior segments than in children with normally sized optic discs. This difference decreases with increasing diameter of the measurement circle. The qualitative comparison of pRNFL thickness profiles (Appendix A.2) of all groups shows a minimal decrease in pRNFL thickness at the temporal inferior RNFL maxima, whereas the LOD-A group also tends to a slight decrease at the temporal superior RNFL maximum.

Table 3 summarizes the differences in pRNFL change between the groups using mixed models with generalized estimated equations (GEE). Adult large optic discs were associated with a significant decrease in pRNFL thickness at follow-up in the global assessment (β = −1.21 µm, *p* = 0.006) and the nasal (β = −1.61 µm, *p* = 0.002) and nasal superior segments (β = −1.35 µm, *p* = 0.046) (Table 3).

### 3.1. Automated Classification Algorithm

The automated OCT-based classification algorithm revealed the highest number of classification changes in the LOD-C group, closely followed by the LOD-A group (Table 4). While the LOD-A group showed a predominance of deteriorations (within normal limits/WNL → borderline/BL or borderline/BL → outside normal limits/ONL), the LOD-C group exhibited slightly more improvements (ONL → BL or BL → WNL). Changes in the NOD-C group were half as frequent as in the groups with large optic discs.

### 3.2. Variance Analysis

**Figure 2:**
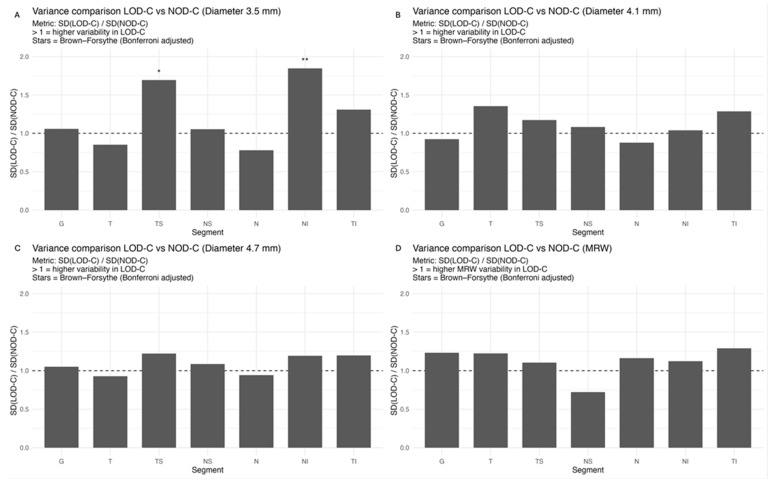
Ratio of RNFL (**A**–**C**) and MRW (**D**) change variability between LOD-C and NOD-C across peripapillary segments. Bars show SD(LOD-C)/SD(NOD-C). A ratio > 1 indicates higher inter-individual variability in the LOD-C group. Asterisks denote significant differences based on the Brown–Forsythe test (Bonferroni-adjusted; * < 0.05, ** < 0.01). Abbreviations: G, global; T, temporal; TS, temporal superior; NS, nasal superior; N, nasal; NI, nasal inferior; TI, temporal inferior.

The comparison of variances using the Brown–Forsythe test and Bonferroni correction revealed a significantly higher variance of pRNFL change in the superotemporal and inferonasal segments in children with large optic discs than in children with normal-sized optic discs at the 3.5 mm measurement circle (Figure 2). This fluctuation was neither present at the 4.1 or 4.7 mm circles nor in the BMO-MRW measurement.

### 3.3. Incident Glaucoma Cases

During the median follow-up period of 1.4 years, none of the discs of LOD-C or NOD-C progressed to glaucoma; however, four optic discs in the LOD-A group showed glaucomatous conversion. One pediatric large optic disc and two adult large optic discs were classified as glaucoma suspects.

## 4. Discussion

Large optic discs in children are commonly diagnosed as glaucoma suspects due to their large cup. In this study, we followed up pediatric large optic discs for a median of 1.4 years and compared them with normal-sized optic discs in children and adult large optic discs. The examinations proved a stable global pRNFL thickness in all three groups and underscore the importance of longitudinal follow-ups in glaucoma suspect optic discs to avoid unnecessary antiglaucomatous treatment in early years of life.

Previous studies reported a higher susceptibility for glaucoma or glaucoma progression in eyes with large optic discs, mediated by an increased displacement of the lamina cribrosa [9,17]. An optic disc analysis using the finite element method (FEM) found supporting results with increasing IOP-related mechanical stress (defined as force/cross-sectional area) in larger optic discs [10]. On the other hand, intraindividual comparisons did not reveal a correlation of disc area and mean defect, albeit the analysis was not corrected for paired samples [6]. In our study, none of the pediatric optic discs but four of the adult optic discs progressed to glaucoma. One of the pediatric large optic discs and two of the adult large optic discs were classified as glaucoma suspects according to the glaucoma specialists’ review of OCT, visual field and IOP. The substantial proportion of glaucoma suspects and the observed progressions to manifest glaucoma highlight the importance of regular follow-up examinations. On the other hand, a large proportion of the included cases was referred to our glaucoma center because of glaucoma suspect optic discs, and there might be a selection bias resulting in a falsely high number of conversions to glaucoma. Population-based studies rather than a clinical approach might provide more reliable insights into the relation of optic disc size and glaucoma risk. However, their methodology would demand a cup-to-disc ratio (CDR)-independent approach for diagnosing glaucoma because optic disc size influences CDR and thus glaucoma prevalence as for example used in the ISGEO (International Society for Geographical and Epidemiological Ophthalmology) classification system [7] and enough large optic discs in the sample.

It is a well-known fact that the retinal nerve fiber count decreases with age, with an average decrease of 400 to 7000 axons per year [1,18]. Correspondingly, the pRNFL thickness in adults shows an age-dependent decrease [19,20,21]. An exception are optic discs in children: previous OCT studies in children observed an increase in pRNFL thickness and BMO-MRW and attributed it to the maturation and growth of the papillary capillary network and glial tissue, and the growth of axon calibers [22,23]. pRNFL thickness may increase by as much as 10% during childhood and early adolescence [22]. In the present study, a similar pRNFL increase was observed in the superonasal segments of pediatric optic discs, regardless of optic disc size.

Albeit optic nerve assessment using OCT showed a good repeatability and reproducibility in children [5,24,25], we found indications of measurement inaccuracies of pRNFL thickness and BMO-MRW after a period of one to two years, especially in eyes with large optic discs. As evident from Appendix A.1, the pediatric groups had larger boxplots and thus a higher variability in the change of pRNFL and BMO-MRW as compared to the adult group. The variability of pRNFL change decreased from the innermost to the outermost measurement circle. The comparison of change variances revealed significantly higher variance of pRNFL thickness in the superotemporal and inferonasal segments in children with large optic discs at the 3.5 mm diameter circle. This difference was not prevalent at the 4.1 or 4.7 mm circles. In our former cross-sectional study, we correspondingly found a thicker pRNFL thickness in the inferonasal and superotemporal segments in the LOD-C group as compared to the NOD-C group [4]. This finding may support the hypothesis that large optic discs tend to have a thicker pRNFL due to the closer location of the measurement circles to the optic disc margin and the transition zone, comprising the reorganization of retinal nerve fibers and neuroglial cells before entering the optic nerve canal [26,27]. The measurement in this transition zone, with a high proportion of neuroglial cells combined with a steady reorganization of neuroglia and capillary networks in the growing pediatric eye, may cause unexpected measurement differences in follow-up examinations. This further underscores the importance of considering the outer measurement circles.

The fluctuations of pRNFL thickness measurement also impact the OCT’s automated classification algorithm. In our sample, the algorithm reported class changes (improvements or deteriorations) at a twofold higher frequency in children with large optic discs compared with children with normal-sized discs. Of note, improvements occurred as often as deteriorations. Likewise, adult large optic discs had a similarly high number of changes as compared to pediatric large optic discs. These findings support the notion that optic disc size influences automated OCT progression analysis, being suggestive of an impaired reliability for extraordinarily configured optic discs, potentially independent from age.

In adults with large optic discs, a decrease of global mean pRNFL thickness amounted to −0.58 µm (3.5 mm diameter) with the highest loss in the superotemporal segment, whereas global mean BMO-MRW decreased by −3.94 µm over the median follow-up time of 1 year. A previous systematic review of longitudinal studies found a similar pRNFL decrease of −0.47 µm/year, but a lower BMO-MRW decrease of −1.61 µm/year [21]; another study by Chauhan et al. including 246 white subjects found a lower decrease of pRNFL (−0.21 µm/year) and BMO-MRW (−1.34 µm/year) [28]. The higher decrease in pRNFL and BMO-MRW in the present cohort may be explained by the incident glaucoma cases; furthermore, due to the lacking adult control group, we are not able to exclude that the higher decrease is due to the larger optic disc size in the LOD-C group. On the other hand, the values from Chauhan et al. were adjusted for BMO area and axial length, and had a roughly even age distribution, and thus possibly may differ from our results [28].

Our study had some limitations. First, due to the retrospective character of the study, we were not able to obtain cycloplegic refractive errors, and some baseline characteristics such as visual field examination, axial length or IOP were not available for all cases (depending on the children’s compliance). These values might be skewed or biased, and this may possibly affect the classification as healthy. Second, the control group cannot be considered a truly healthy cohort, since referrals were predominantly based on suspected glaucomatous optic disc morphology or nonglaucomatous conditions (e.g., strabismus). All control eyes demonstrated normal clinical and OCT findings upon expert evaluation by a glaucoma specialist; however, minor deviations from a completely healthy ocular status cannot be entirely excluded, and the number of cases converted to glaucoma may possibly be biased. Third, as we did not expect any glaucomatous progression, we did not include an adult group with normal-sized optic discs; thus, we cannot truly estimate if disc size is associated with a higher glaucoma susceptibility. Further longer-term prospective studies are needed to determine glaucoma risk in large optic discs, especially in children. Lastly, the threshold of 2.5 mm^2^ for large-sized optic discs is not a validated value but historically determined in fundoscopic or Heidelberg retina tomography studies and adopted to OCT-based analyses [2,11,12,13,14,15,16]. The true OCT cut-off value may be estimated to be higher numbers such as 2.8 or 2.9 mm^2^ as reported in other studies [4,29].

## 5. Conclusions

Children with large optic discs presented with stable pRNFL and BMO-MRW and without clinical progression to glaucoma at follow-up. Thus, follow-up examination after one to two years may be a safe method to monitor glaucoma suspect large optic discs in children; however, long-term longitudinal studies with larger sample sizes are needed to evaluate glaucoma risk and to confirm sufficient follow-up intervals. OCT is a helpful tool; however, fluctuations of pRNFL thickness and BMO-MRW appear to be independent of age and may be mainly influenced by structural rather than age-dependent mechanisms in large optic discs, impairing the accuracy of automated classification algorithms. A manual assessment of three different measurement circles can help to appropriately evaluate RNFL changes. Furthermore, individuals with large optic discs may require close surveillance as they might exhibit a higher glaucoma susceptibility.

## Figures and Tables

**Table 1:** Descriptive statistics at baseline.

	LOD-C	NOD-C	LOD-A
Number	85	72	78
Age [years]	11.1 ± 3.1	10.5 ± 3.1	48.3 ± 15.7
Sex (female)	39 (45.9%)	35 (48.6%)	49 (62.8%)
Follow-up time [years]	1.2 [0.9; 2.4]	1.4 [1.0; 2.8]	1.5 [1.0; 2.8]
Visual acuity [LogMAR]	0.03 ± 0.06	0.07 ± 0.2	0.03 ± 0.06
Mean deviation visual field [dB]	3.0 ± 3.0	3.7 ± 3.7	2.7 ± 2.4
Spherical equivalent [D]	−0.5 ± 2.4	−0.1 ± 2.1	−1.0 ± 3.1
Axial length [mm]	23.8 ± 1.3	22.4 ± 4.7	23.4 ± 3.9
Intraocular pressure [mmHg]	16.6 ± 3.2	16.6 ± 3.4	15.0 ± 2.4
BMO area [mm^2^]	2.83 [2.62; 3.0]	2.17 [1.83; 2.32]	2.71 [2.60; 2.85]
Mean global pRNFL thickness [µm]	99.3 ± 11.7	94.4 ± 11.2	96.6 ± 9.8
Mean global BMO-MRW [µm]	279.0 ± 41.7	300.2 ± 66.1	255.0 ± 46.7

Abbreviations: LOD-C, large optic discs in children; NOD-C, normally sized optic discs in children; LOD-A, large optic discs in adults; pRNFL, peripapillary retinal nerve fiber layer; BMO-MRW, Bruch’s membrane opening-minimum rim width.

**Table 2:** Mean differences [95% CI] in µm in the peripapillary sectors (simplified Garway Heath schemes; The icon in the top left cell indicates the segments: global (G), center; temporal (T), left; temporal superior (TS), top left; nasal superior (NS), top right; nasal (N), right; nasal inferior (NI), bottom right; temporal inferior (TI), bottom left). Unadjusted statistical significance of paired *t*-test (baseline vs. 1-year follow-up) is indicated by stars: * *p* < 0.05; ** *p* < 0.01; *** *p* < 0.001 in paired *t*-tests. Grey stars indicate nominal statistical significance; black stars indicate statistical significance after Bonferroni’s correction.

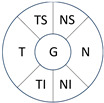	LOD-C	NOD-C	LOD-A
pRNFL 3.5 mm	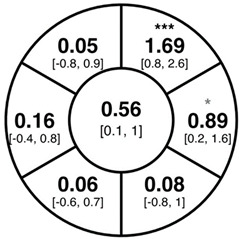	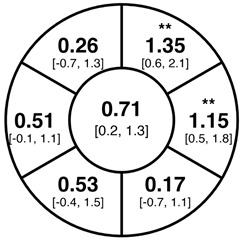	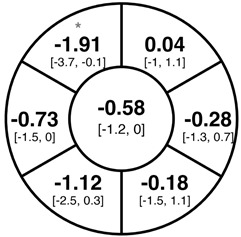
pRNFL 4.1 mm	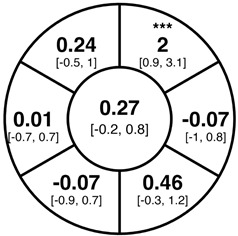	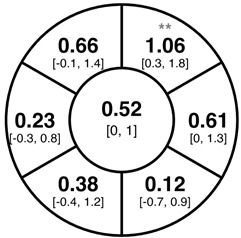	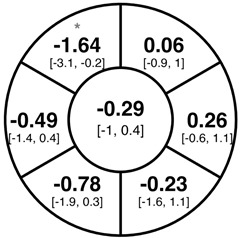
pRNFL 4.7 mm	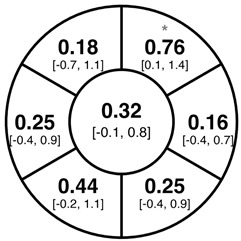	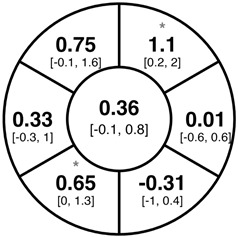	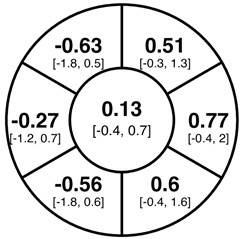
BMO-MRW	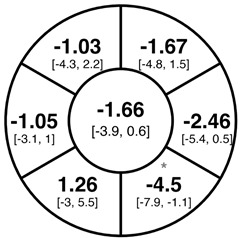	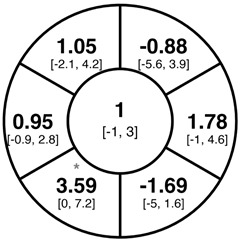	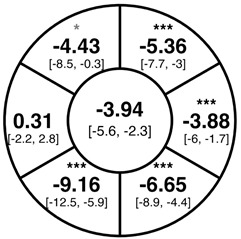

Abbreviations: LOD-C, large optic discs in children; NOD-C, normally sized optic discs in children; LOD-A, large optic discs in adults; pRNFL, peripapillary retinal nerve fiber layer; BMO-MRW, Bruch’s membrane opening-minimum rim width.

**Table 3:** Mixed models with generalized estimated equations (GEE) to compare changes in pRNFL thickness between baseline and follow up of LOD-C vs. NOD-C and LOD-C vs. LOD-A (3.5 mm circle).

	Beta	95% CI	*p*
**Δ Global pRNFL**			
LOD-C	reference		
NOD-C	0.10	−0.74; 0.95	0.93
LOD-A	−1.21	−2.07; −0.35	**0.006**
**Δ Temporal pRNFL**			
LOD-C	reference		
NOD-C	0.37	−0.50; 1.24	0.41
LOD-A	−0.61	−1.51; 0.29	0.19
**Δ Temporal superior pRNFL**			
LOD-C	reference		
NOD-C	0.24	−1.18; 1.65	0.74
LOD-A	−1.40	−3.21; 0.41	0.13
**Δ Nasal superior pRNFL**			
LOD-C	reference		
NOD-C	−0.33	−1.58; 0.92	0.61
LOD-A	−1.35	−2.68; −0.02	**0.046**
**Δ Nasal pRNFL**			
LOD-C	reference		
NOD-C	0.24	−0.81; 1.28	0.66
LOD-A	−1.61	−2.62; −0.59	**0.002**
**Δ Nasal inferior pRNFL**			
LOD-C	reference		
NOD-C	−0.04	−1.42; 1.48	0.96
LOD-A	−0.81	−2.21; 0.59	0.26
**Δ Temporal inferior pRNFL**			
LOD-C	reference		
NOD-C	0.36	−0.96; 1.69	0.59
LOD-A	−1.40	−2.84; 0.03	0.06

Abbreviations: LOD-C, large optic discs in children; NOD-C, normally sized optic discs in children; LOD-A, large optic discs in adults; pRNFL, peripapillary retinal nerve fiber layer.

**Table 4:** Evaluation of the changes in OCT’s automated classification algorithm. Deteriorations (WNL → BL or BL → ONL) and improvements (ONL → BL or BL → WNL) were counted for each group.

Group	Total Changes	Deteriorations	Improvements	Most Changed Segment
LOD-C	42	19	23	G
NOD-C	20	9	11	G
LOD-A	42	31	11	N

Abbreviations: LOD-C, large optic discs in children; NOD-C, normally sized optic discs in children; LOD-A, large optic discs in adults; G, global; N, nasal.

**Figure A1:**
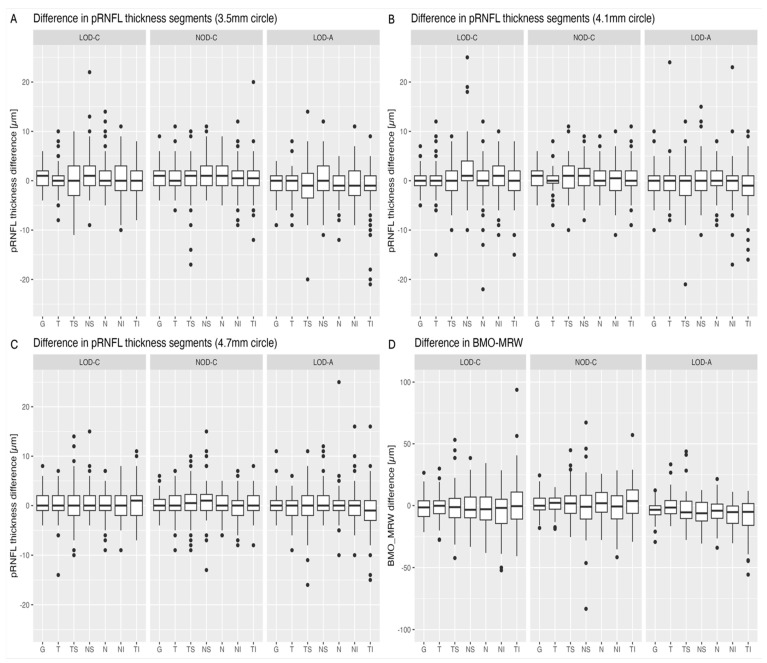
Change of pRNFL thickness (**A**–**C**) and BMO-MRW (**D**) for each Garway Heath sector after 1 year. Abbreviations: G, global; T, temporal; TS, temporal superior; NS, nasal superior; N, nasal; NI, nasal inferior; TI, temporal inferior; LOD-C, large optic discs in children; NOD-C normally-sized optic discs in children; LOD-A, large optic discs in adults.

**Figure A2:**
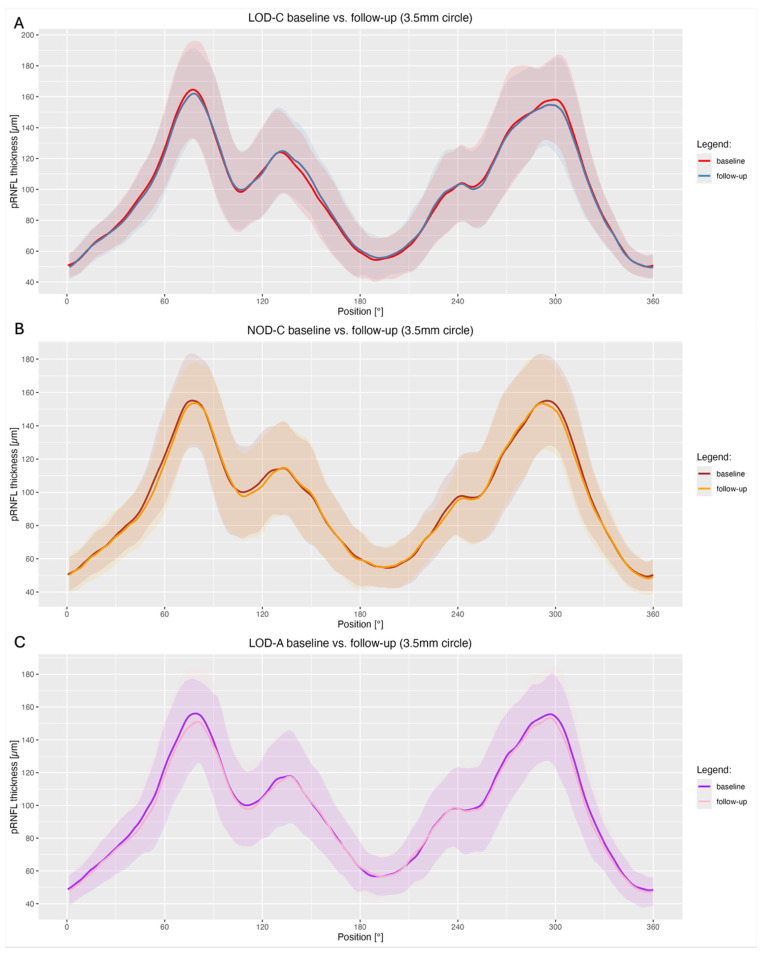
pRNFL profiles (3.5 mm circles) of baseline and follow-up examination for LOD-C (**A**), NOD-C (**B**) and LOD-A (**C**).

## Data Availability

Data is available upon request.

## Abbreviations

The following abbreviations are used in this manuscript:

BL	Borderline
BMO	Bruch membrane opening
BMO-MRW	Bruch membrane opening-minimum rim width
CI	Confidence interval
CDR	Cup-to-disc-ratio
FEM	Finite element method
G	Global
GEE	Generalized estimating equations
IOP	Intraocular pressure
ISGEO	International Society for Geographical and Epidemiological Ophthalmology
IQR	Interquartile range
LOD-A	Large optic discs in adults
LOD-C	Large optic discs in children
N	Nasal
NI	Nasal inferior
NOD-C	Normal-sized optic discs in children
NS	Nasal superior
OCT	Optical coherence tomography
ONL	Outside normal limits
pRNFL	Peripapillary retinal nerve fiber layer
SD	Standard deviation
T	Temporal
TI	Temporal inferior
TS	Temporal superior
WNL	Within normal limits
